# Lexicon dataset for the Hausa language

**DOI:** 10.1016/j.dib.2024.110124

**Published:** 2024-02-01

**Authors:** Idi Mohammed, Rajesh Prasad

**Affiliations:** Computer Science Department, African University of Science and Technology, Abuja, Nigeria

**Keywords:** Lexicon-based, Sentiment analysis, Data augmentation, Low-resource languages, Social media

## Abstract

This paper presents a comprehensive augmented lexicon sentiment analysis dataset for the Hausa language. The dataset was created by adopting words and phrases from a Hausa Language dictionary and then using the data augmentation method to expand the quantity of the dataset. The researchers manually annotated each phrase/sentence with positive, negative, or neutral polarity. The dataset consists of 14,663 rows, with 4,154 positives, 4,310 negatives, and 6,199 neutrals. The dataset is valuable because it contributes to the available resources for sentiment analysis, especially for Hausa, which is a low-resource language. The dataset will benefit researchers in sentiment analysis who want to develop a model to analyze Hausa posts on social media or product reviews in the Hausa language.

Specifications Table

**Table:** 

Subject	Computer Science
Specific subject area	Sentiment analysis dataset for low-resource languages
Data format	Raw
Type of data	Table
Data collection	Data was acquired from an English/Hausa dictionary: Kamus Na Hausa Zuwa Turanci [1]
Data source location	https://drive.google.com/file/d/1OttyNIkxyfFYrWoIF2L_41J4TTR4KRcg/view?usp=sharing
Data accessibility	Repository name: Hugging Face Data Hub Data identification number: 10.57967/hf/1541
Related research article	None

## 1 Value of the Data

• These datasets are important because they contribute to the available resources for sentiment analysis, especially for Hausa, which is a low-resource language [2].
• The datasets will benefit researchers in sentiment analysis who want to train their models to analyze Hausa posts on social media, product review for Hausa, or any form sentiment analysis that has to do with Hausa language.
• The dataset is useful for researchers in NLP who want to develop a sentiment analysis dataset for low-resource languages other than Hausa using Hausa as a source language, especially for languages in sub-Sahara Africa having similar dialects with Hausa.

## 2 Background

The development of a Lexicon Dataset for the Hausa Language originated from a pressing requirement to facilitate and augment research and applications in NLP on this significant West African language. Hausa is a language that exhibits extensive usage across the region, boasting a substantial speaker population of 80 million people. Moreover, it serves as a prominent lingua franca in Nigeria, Niger Republic, and many neighbouring nations.

The rationale for creating this dataset is to tackle the limited availability of extensive linguistic resources for the Hausa language. This shortage presents a notable challenge in the advancement of NLP techniques and technology specifically designed for Hausa-speaking people. The primary objective in creating the dataset was to offer a comprehensive lexical resource to researchers, developers, and language aficionados, which could be utilized in various NLP applications.

There is a similar study [3],which contributed for the development of lexicons for the Hausa language with 1000 negatives, 1014 positives, and with zero neutrals. The dataset provided is smaller in size when compared with our submission and the submission lacks neutrality, which according to [4], is crucial for discerning between positive and negative. Another contributor [5] provided sentiment analysis dataset with 14,000+ annotated comments obtained from social media platforms with 4574 negatives, 4687 positives, and 4912 neutrals. With millions of social media users who communicate in Hausa language, NLP model trained with 14,000 social media comments will be less capable in detecting polarity as most of the comments are influenced by topic of discussion which are timely elapse. Example, some of the comments are related to Covid-19 which is less attractive as a topic of discussion in social median at present.

This research develops lexicon-based approach which drive text polarity from words or phrases polarity that occur in it [6]. Enhance-lexicon leverage the advantage of lexicon-based approaches and the corpus-based approach due to augmentation. This advantage will give more accuracy to models trained with our dataset that is enhance-lexicon than models trained with social media comments for the Hausa language as started in [5].

Researchers interested in using our dataset can access directly via Hugging Face datasets hub [7] or for researchers who are using transformers can load directly with python code or any capable programming languages using dataset = load_dataset(“mangaphd/hausa_aug_lex”).

## 3 Data Description

The data is stored in comma-separated values (csv) file format. It is a dataset consisting of 14,663 unique records [7]. Every data entry is classified into one of three categories: positive, negative, or neutral. English translation is provided for each row. Table 1 presents the descriptions corresponding to the names of each column. There is a total of 4154 positives, 4310 negatives, and 6199 neutrals annotated augmented lexicons. Additionally, the datasets included in our study consist of bigrams and higher-order n-grams.

**Table 1:** Columns in dataset and their description.

Column Name	Description
S/No	Unique identification number for each word.
Hausa	consist of word or phrase in Hausa Language.
Polarity	associated polarity of text in Hausa column.
English Meaning	Consist of translated meaning from Hausa to English.

The significance of these datasets lies in their contribution to the existing resources for sentiment analysis, particularly for Hausa, a language that is considered to have few resources accessible for such research [2]. The datasets can be used for the purpose of NLP Applications. These datasets are expected to be advantageous for academics engaged in sentiment analysis, particularly those interested in constructing a model to evaluate Hausa postings on social media platforms or product review. The dataset plays a significant role in the advancement of Natural Language Processing systems for research purposes in Hausa, which is considered a language with limited resources.

## 4 Experimental Design, Materials and Methods

In this study, we used Hausa words provided in [1]. Creating a sentiment lexicon manually by annotating words with their respective sentiment is a scalable solution for small corpora [8]. Consequently, an automated approach that employs a corpus or dictionary is a more desirable alternative [8]. In this research we adopted Data Augmentation Techniques(DAT) by [9] to build our dataset. Lexicons that are constructed by human experts typically exhibit higher levels of accuracy than the machine method [6] especially in low resources languages. In the first place, we identify list of words from the Hausa language dictionary by [1]. In each word, we manually performed synonym replacement, random insertion, random swap, and random deletion to create sets of positives and negatives sentences or phrases resulting in 14,663 datasets. Annotation of augmented test has been carried out for each phrase immediately after completion. Table 2 shows the method used for data augmentation with examples.

**Table 2:** Data augmentation techniques examples.

DAT Method	Description	Example in positive case	Example in negative case
Synonym Replacement	Select **n** words and replace each with one of their synonyms.	**Fitattune** replaced with **zababbune**	**Tulin shara** replaced it with **Tarin shara**
Random Insertion	Find a word in the phrase or sentence, and then insert its synonym at a random position. This is repeated **n** times.	**Mai bajinta** inserted **Mai bajinta sosai**	**Murdiyane sosai** inserted **Murdiyane sosai da mugunta**
Random Swap	Choose two words and switch their positions at random within the phrase or sentence. This procedure is repeated **n** times.	**Fari kyakkyawa** after swapping **kyakkyawa fari**	**Karyayye mugu** after swapping **mugu karyayye**
Random Deletion	Randomly remove each word from the phrase or sentence with probability **p**.	**Inganta harkar tsaro** after deletion **inganta tsaro**	**Marandadi matuka** after deletion **marandadi**

The second phase of dataset creation employed the services of five experts in Linguistics to proofread each record and validate the annotation. The experts corrected some spellings, confirm polarity class, and re-adjusted sentences to reflect the most used words and phrases among Hausa speakers.We assessed annotator agreement reliability using Cohen's kappa coefficient by [10]. Firstly, five languages specialists validated 14,663 rows. Annotators work separately. After the task, we tested if phrase polarity was accepted if at least one alternative was chosen, and the inter-agreement score was over 0.65. The final aspect of the work produces the English meaning after translation from Hausa to English. The translation was done by experts in Linguistics who proofread the dataset.

## Limitations

Our dataset is limited to Lexicon-based NLP models. Our dataset is not appropriate for the training of data-driven models.

## Ethics Statement

The current work does not involve human subjects, animal experiments, or any data collected from social media platforms.

## CRediT authorship contribution statement

**Idi Mohammed:** Writing – original draft. **Rajesh Prasad:** Supervision, Validation, Writing – review & editing.

## Data Availability

hausa_aug_lex (Original data) (Hugging face data hub).

## Appendix Supplementary materials

Image, application 1
